# Fabrication of Ga_2_O_3_ Schottky Barrier Diode and Heterojunction Diode by MOCVD

**DOI:** 10.3390/ma15238280

**Published:** 2022-11-22

**Authors:** Teng Jiao, Wei Chen, Zhengda Li, Zhaoti Diao, Xinming Dang, Peiran Chen, Xin Dong, Yuantao Zhang, Baolin Zhang

**Affiliations:** State Key Laboratory on Integrated Optoelectronics, College of Electronic Science and Engineering, Jilin University, Qianjin Street 2699, Changchun 130012, China

**Keywords:** Ga_2_O_3_, Schottky barrier diodes, heterojunction, MOCVD

## Abstract

In this article, we reported on a Ga_2_O_3_-based Schottky barrier diode and heterojunction diode from MOCVD. The Si-doped n-type Ga_2_O_3_ drift layer, grown by MOCVD, exhibited high crystal quality, flat surfaces, and uniform doping. The distribution of unintentional impurities in the films was studied. Then nickel Schottky barrier diode and p-NiO/n-Ga_2_O_3_ heterojunction diode were fabricated and measured. Without any electric field management structure, the Schottky barrier diode and heterojunction diode have specific resistances of 3.0 mΩ·cm^2^ and 6.2 mΩ·cm^2^, breakdown voltages of 380 V and 740 V, thus yielding power figures of merit of 48 MW·cm^−2^ and 88 MW·cm^−2^, respectively. Besides, both devices exhibit a current on/off ratio of more than 10^10^. This shows the prospect of MOCVD in power device manufacture.

## 1. Introduction

In recent years, the potential of Ga_2_O_3_ in power devices has been paid more attention to due to its high band gap of 4.9 eV, breakdown electric field strength of 8.0 MV/cm, and lower conduction losses [1,2,3,4,5]. The realizations of Ga_2_O_3_ materials in crystal growth [6,7,8], film preparation [9,10,11,12,13], and doping control [14,15,16,17] provide conditions for high-performance Ga_2_O_3_ devices. Currently, vertical structure Ga_2_O_3_-based Schottky barrier diodes (SBDs) [4,5,18,19,20], heterojunction diodes (HJDs) [21,22,23,24,25] have been widely reported due to their advantages in process complexity, thermal management, and current flow capability. 

In semiconductor devices, a high-quality film with stable electrical properties is an important factor in ensuring the device’s performance. Ga_2_O_3_ films can be grown by molecular beam epitaxy (MBE) [26,27], low-pressure chemical vapor deposition (LPCVD) [14], halide vapor phase epitaxy (HVPE) [28,29], mist-chemical vapor deposition (mist-CVD) [13], and metal-organic chemical vapor deposition (MOCVD) [12,15]. Currently, HVPE is the dominant method for Ga_2_O_3_ epitaxial wafers due to faster growth rates and lower carbon impurity. However, since the fast growth rate will lead to contamination and a rough surface of the film, chemical mechanical polishing (CMP) is necessary to achieve a smooth surface. At the same time, other impurities will be introduced into the film during the CMP [16]. Compared with HVPE, the growth rate of MOCVD is slower, but the surface of the Ga_2_O_3_ film tends to show a uniform step flow mode, making epitaxial wafers unnecessary to polish. In addition, MOCVD also has advantages in precise doping and low background carrier concentration, which makes MOCVD have great potential in growing Ga_2_O_3_ films [16,18]. In the current reports, the fabrications of Ga_2_O_3_ SBDs and HJDs are mostly based on HVPE Ga_2_O_3_ epitaxial wafers [4,5,19,20,24]. However, there are few reports on Ga_2_O_3_ power diodes from MOCVD [18].

In this paper, the homoepitaxy of the Ga_2_O_3_ drift layers was performed on the (001) Ga_2_O_3_ substrate by MOCVD, and the nickel SBD and p-NiO/n-Ga_2_O_3_ HJD were fabricated. The devices exhibit relatively low specific on-resistances **(R_on, sp_)** and high breakdown voltages **(BVs)** at a thickness of 5.0 μm without any edge termination. This performance is comparable to those of Ga_2_O_3_ devices fabricated by HVPE, demonstrating that MOCVD Ga_2_O_3_ is promising for high-performance vertical power devices.

## 2. Materials and Methods

The growth of β-Ga_2_O_3_ film was performed on the double-sided polished (001) β-Ga_2_O_3_ substrates. The substrate was first cleaned with toluene, acetone, ethanol, and deionized water. Then the n-type Ga_2_O_3_ film was grown under the conditions of chamber pressure and substrate temperature of 40 mbar and 750 °C, respectively. The Ga_2_O_3_ film consists of a 200 nm buffer layer and a 5.0 μm drift layer, with electron concentrations of 5.0 × 10^18^ cm^−3^ and 3.5 × 10^16^ cm^−3^, respectively. The MOCVD (Emcore D180) uses trimethylgallium (TMGa, 6N) and high-purity oxygen (6N) as the sources, having a growth rate of ~1.0 μm/h. The films were doped with silane (SiH_4_, diluted in nitrogen, 50 ppm). Argon (6N) works as a carrier gas for TMGa saturated vapor and SiH_4_. The p-NiO film was grown by magnetron sputtering (JZCK-IVB) at room temperature, using a NiO: Li target with a Li mass fraction of 5% to improve the conductivity. The deposition speed of NiO films was 130 nm/h. More details on the preparation and characterization of NiO films can be found in our prior study [30,31]. The device fabrication started with the deposition of Ti (20 nm)/Au (100 nm) electrodes on the backside of the substrate, followed by rapid thermal annealing (RTA) at 500 °C in an N_2_ atmosphere for 1 min to ensure good ohmic contact. Then Ni (50 nm)/Au (100 nm) was patterned by a liftoff process on the top with a diameter of 150 μm.

The films were characterized by X-ray diffraction (XRD, Rigaku, Ultima IV, Tokyo, Japan, CuKα radiation, λ = 1.54 Å), atomic force microscopy (AFM, Veeco, PlainView, NY, USA), and field emission scanning electron microscopy (FESEM, JOEL, JSM-7900, Tokyo, Japan). X-ray photoelectron spectroscopy (XPS, Thermo Fisher Scientific, ESCALAB250, Waltham, MA, USA) and **secondary ion mass spectroscopy (SIMS, Cameca, IMS 4f auto, Gennevilliers, France)** were used to characterize the state and distribution of elements on the film surface. Device performance was measured and analyzed by Agilent B2912A and Keithley 2470, which were connected to the probe system (Lake Shore Cryotronics, Model CRX-6.5K, Westerville, OH, USA).

## 3. Results

### 3.1. Film Characterization

**Figure 1:**
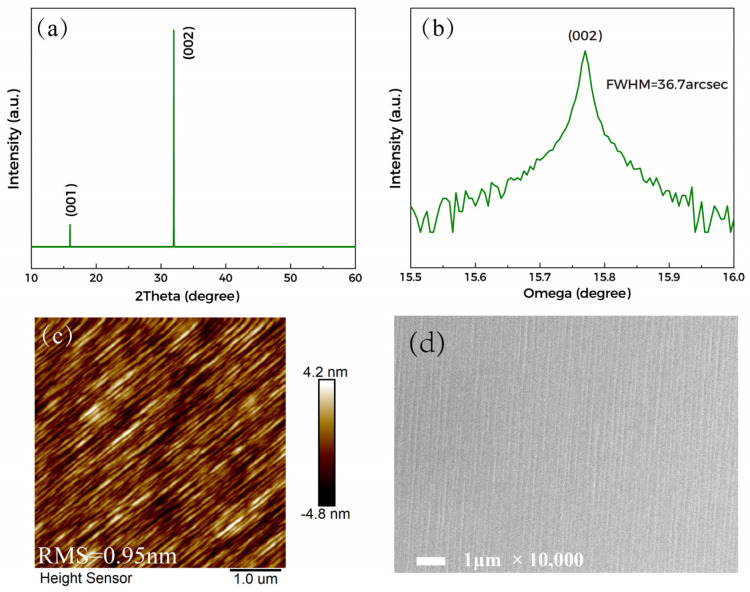
Results of Ga_2_O_3_ characterization; (**a**) XRD 2theta−omega patterns; (**b**) XRD rocking curve of (002) diffraction plane; (**c**) AFM; (**d**) FESEM.

The characterization results of Ga_2_O_3_ films are shown in Figure 1. From the XRD in Figure 1a,b, it can be found that the Ga_2_O_3_ film exhibits a strong preferred orientation along the <001> direction. The full width at half maximum (FWHM) of the rocking curve of the (002) diffraction plane is 36.7 arcsec, basically close to the 28.6 arcsec of the substrate, indicating low dislocation density. From the FESEM and AFM images shown in Figure 1c,d an obvious step-flow growth pattern with consistent direction can be seen on the film surface. Besides, the film surface is smooth and uniform, and the root mean square (RMS) roughness is 0.95 nm. These characterizations indicate the high crystal quality of the films.

**Figure 2:**
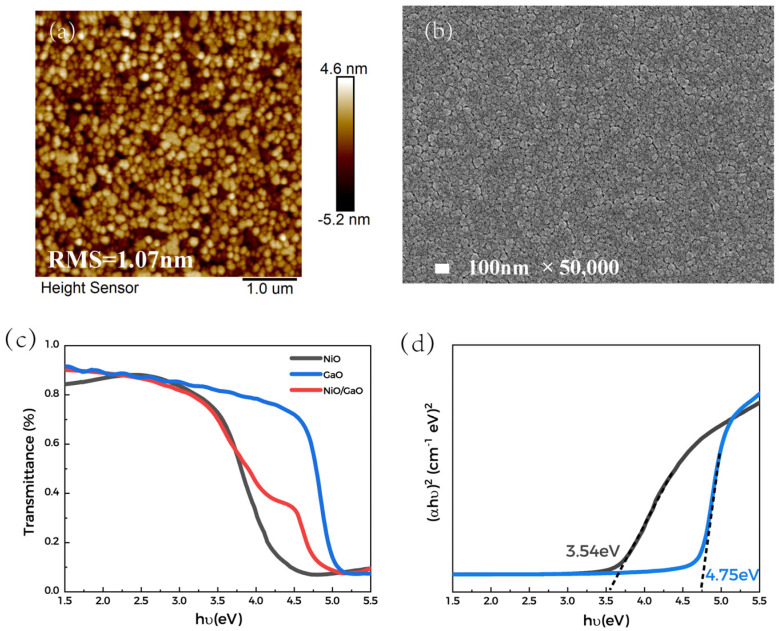
Results of NiO characterization; (**a**) AFM; (**b**) SEM; (**c**) Transmission spectra; (**d**) Tauc plot.

Figure 2 shows the characterization of NiO films. Figure 2a,b shows the surface of NiO film grown on Ga_2_O_3_ films. The NiO film has a dense surface with an RMS roughness of about 1.07 nm. The optical properties of NiO grown on the sapphire and Ga_2_O_3_ wafers were characterized by transmittance spectroscopy, as shown in Figure 2c. All the films exhibited a high transmittance rate (>80%) in the energy range of 1.5–3.5 eV. Figure 2d is the Tauc plot. The band gaps of Ga_2_O_3_ and NiO were obtained by linear extrapolation to be 4.75 eV and 3.54 eV, respectively. Besides, by Hall measurement at room temperature, the NiO film exhibited a resistivity of 5.0 Ω·cm with a hole concentration and mobility of 5 × 10^18^ cm^−3^ and 0.47 cm^2^/V·s, respectively.

**Figure 3:**
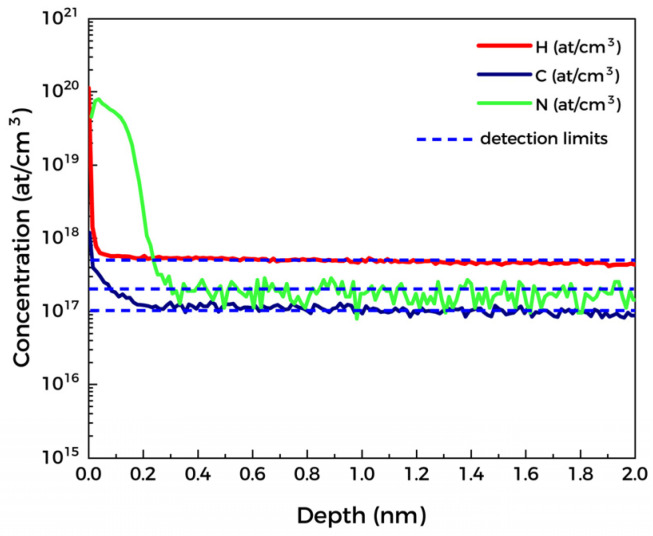
SIMS on the film surface.

To investigate the impurity contamination on the film surface and the distribution of unintentional doping elements, the SIMS was performed on N, H, and O, as shown in Figure 3. It can be found that the content of C and H is relatively high on the film surface. H is present only in the depth range of ~20 nm on the surface, while the penetration depth of C and N is ~0.2 μm. Since the content of all three elements decays rapidly to the detection limit after 0.2 μm away from the film surface, it can be inferred that the impurity elements originate from the adsorption of impurities in the environment and contamination. However, surface impurities will significantly change the surface state of the film, including interface state density, charge accumulation, and surface barrier, thereby affecting the performance of the device [19]. Therefore, the exposure time to air needs to be minimized to reduce the adsorption of impurities.

**Figure 4:**
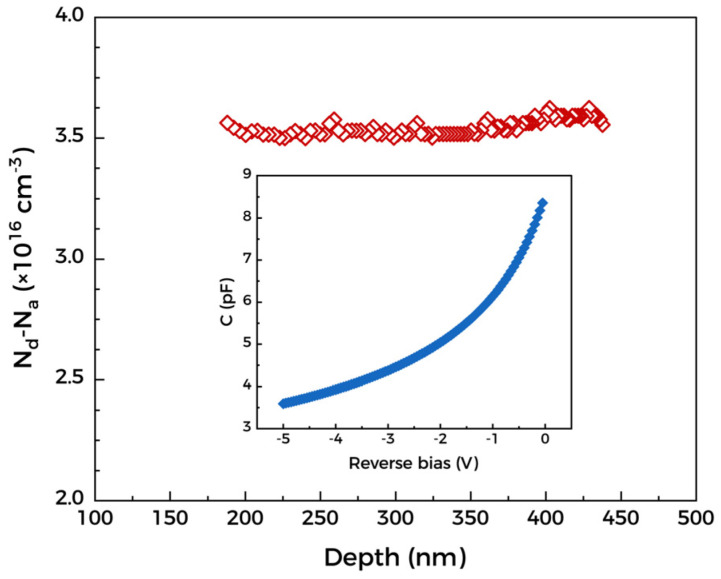
C-V characteristics measured under 500 kHz.

The electron concentration of the Ga_2_O_3_ drift layer was characterized by the capacitance-voltage (C-V) measurement at room temperature under 500 kHz due to the conductive substrate. The plot of electron concentration with depth is shown in Figure 4. It can be seen intuitively that the values of N_d_-N_a_ are all stable at around 3.5 × 10^16^ cm^−3^ at different depths, which indicates the superiority of MOCVD in doping accuracy. The C-V relationship is shown in the inset. In the voltage range of 0 V to −5 V, the depletion layer ranges from 200 nm to 450 nm and is not completely depleted [32]. In addition, the MOCVD-grown Ga_2_O_3_ films with this electron concentration value have an electron mobility of ~140 cm^2^/V·s, according to our prior work [33].

### 3.2. Devices Measurement

**Figure 5:**
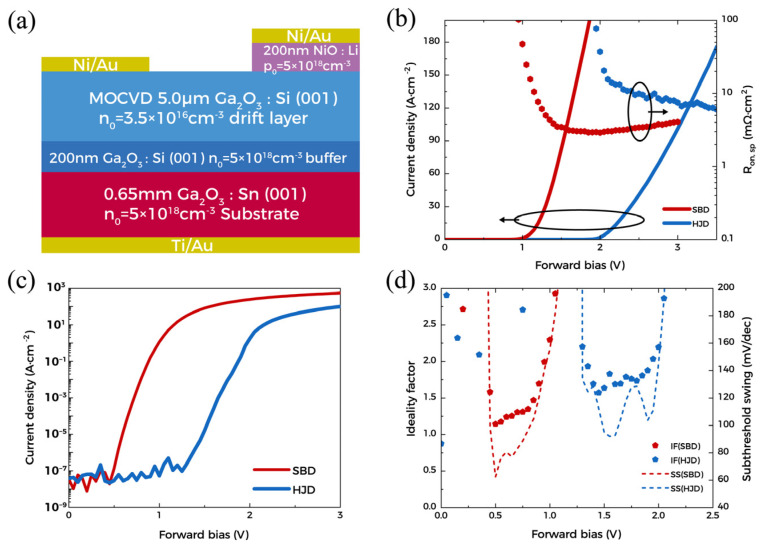
(**a**) Cross-section schematic of devices; (**b**) Linear scale J–V and R_on, sp_; (**c**) Log scale J–V plot; (**d**) Ideality factor and subthreshold swing.

Figure 5 shows the structure and the forward characteristic of the devices. From Figure 5b, the turn-on voltages (V_on_) of SBD and HJD are about 1.0 V and 2.0 V, respectively. The R_on, sp_ of the SBD and HJD are 3.0 mΩ·cm^2^ and 6.2 mΩ·cm^2^, obtained by the derivative of the curve. Higher electron concentration and lower drift layer thickness enable the device to achieve low R_on, sp_. From the logarithmic scale forward characteristic curve in Figure 5c, both SBD and HJD exhibit a current on/off ratio of more than 10^10^ at 3 V. Besides, the ideality factor and subthreshold swing (SS) of a diode can be calculated according to the following relationship:

(1) $$n=\frac{qdV}{kTd\left(lnJ\right)}$$

(2) $$SS=\frac{dV}{d\left(logJ\right)}$$

where *J* represents the current density, *k* is the Boltzmann constant, and *n* is the ideality factor [34]. The ideality factor and *SS* are shown in Figure 5d. In the near-linear barrier-controlled region, the ideality factor of SBD can be maintained at about 1.1~1.2, suggesting a dominant thermionic emission model [35]. In contrast, the ideality factor of HJD is stably maintained around 1.7, which has been reported to be related to interface recombination caused by large lattice mismatch [36,37]. The minimum *SS* of SBD and HJD fluctuate, ranging from ~63 mV to ~90 mV, respectively. A current change of 6 orders of magnitude can be achieved before the SS increases to 200 mV, which indicates a low density of trap states at the interface. In addition, the average SS of SBD is significantly lower than that of HJD, indicating a faster switching speed.

**Figure 6:**
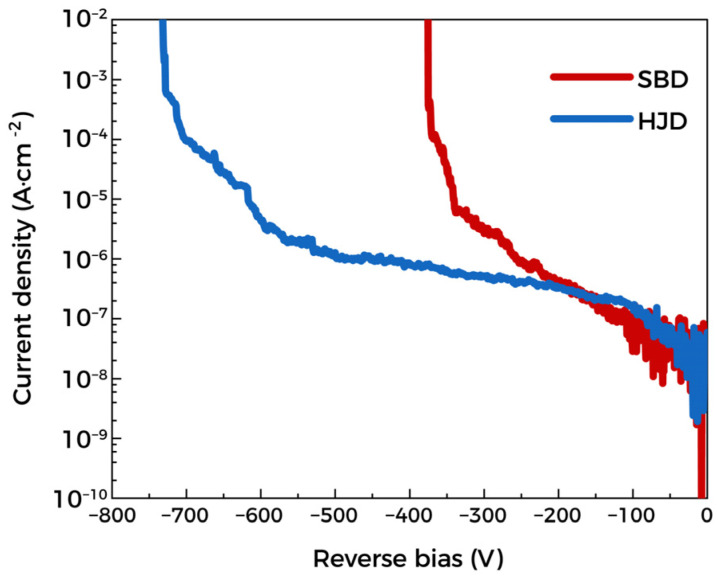
Reverse characteristics of Ga_2_O_3_ SBD and HJD.

Figure 6 is the breakdown characteristic of devices. The BVs of SBD and HJD are 380 V and 740 V, thus yielding to P-FOMs of 48 MW·cm^−2^ and 88 MW·cm^−2^, respectively. Through the one-dimensional Poisson equation and the assumption of the planar junction, the SBD and HJD depletion width is calculated to be 3.4 μm and 4.8 μm when the BVs are applied. Since 3.4 μm and 4.8 μm are smaller than the drift layer thickness, this indicates that the devices are non-punch-through, mainly caused by the higher electron concentration of the film and electric field crowding at the anode edge. Furthermore, since the film surface is exposed to air for a long time and has not been treated, the adsorption of impurities can also cause an increase in devices leakage current. Etching away the Ga_2_O_3_ film with a thickness of ~200 nm from the surface by dry etching and then repairing the surface damage will greatly improve the ideality factors and BVs of the devices [19].

**Figure 7:**
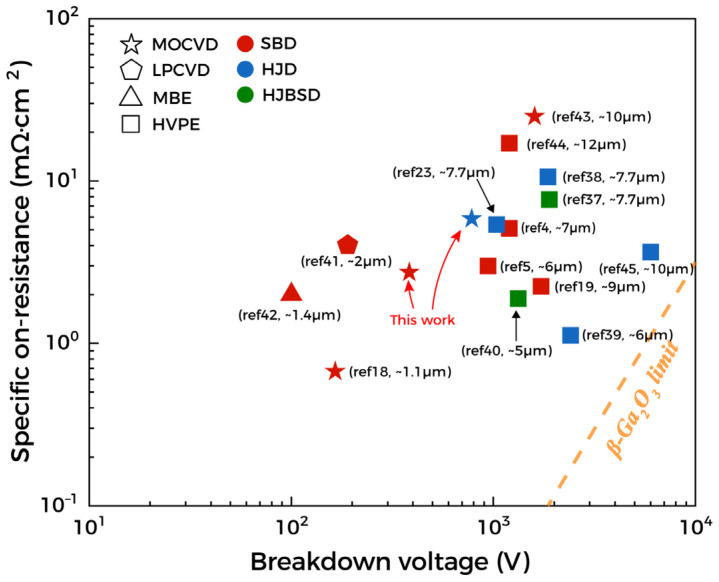
Plot of the specific on-resistance and breakdown voltage for reported Ga_2_O_3_ power diodes [4,5,18,19,23,37,38,39,40,41,42,43,44,45].

The benchmark relationship between Ga_2_O_3_ BV and R_on, sp_ is shown in Figure 7, which also lists the prior reports on three types of different devices, including SBDs, HJDs, and heterojunction barrier Schottky diodes (HJBSDs) fabricated by different growth methods. From the figure, although there are few vertical power diodes based on MOCVD, according to existing research, the performance of diodes from MOCVD has achieved higher P-FOM with thinner thickness [18]. In this work, the diodes have lower BVs due to a larger leakage current caused by a high electric field. Therefore, by appropriately increasing drift layers thickness, reducing the electron concentration, and adding edge terminations or surface electric field management structures, the performance of the device will be further improved. This shows the promise of MOCVD in Ga_2_O_3_ power devices.

## 4. Discussion

The homoepitaxy of n-Ga_2_O_3_ films on (001) substrate was achieved. Various characterizations demonstrated the films’ high crystal quality and stable electrical properties. On this basis, Ga_2_O_3_ SBD and HJD were fabricated and measured. In terms of the forward characteristics, the R_on, sp_ of SBD is 3.0 mΩ·cm^2^, which is smaller than that of HJD. Besides, HJD exhibits a large ideal factor because of the complex current transport mechanisms caused by the lattice mismatch and the adsorption of impurities at the interface. In terms of reverse characteristics, the BVs of the SBD and HJD are 380 V and 740 V, respectively, which indicates that HJD has significantly better blocking characteristics than SBD. The P-FOM of the SBD and HJD are 48 MW·cm^−2^ and 88 MW·cm^−2^, comparable to reported SBD and HJD with the same structure. However, the high electric field at the anode edge will result in large leakage currents at high reverse bias voltages. Therefore, appropriate edge termination or electric field management structures, such as implanted edge termination, field plates, field limiting rings, etc., will increase device BV further.

## 5. Conclusions

SBD and HJD based on high-quality homoepitaxial Ga_2_O_3_ films from MOCVD were reported. Without any electric field management structure, the SBD achieves a R_on, sp_ of 3.0 mΩ·cm^2^ and a BV of 380 V, compared to 6.2 mΩ·cm^2^ and 740 V for the HJD. Due to the relatively high electron concentration of the film and the electric field concentration effect at the anode edge of the devices, the devices are non-punch-through. Therefore, appropriately reducing the electron concentration of the films while the field management structure or punch-through design is the next research direction.

## Data Availability

The data presented in this study are available on request from the corresponding authors.
